# Approach to the diagnosis of drug hypersensitivity reactions: similarities and differences between Europe and North America

**DOI:** 10.1186/s13601-017-0144-0

**Published:** 2017-03-13

**Authors:** M. J. Torres, A. Romano, G. Celik, P. Demoly, D. A. Khan, E. Macy, M. Park, K. Blumenthal, W. Aberer, M. Castells, A. Barbaud, C. Mayorga, P. Bonadonna

**Affiliations:** 1 Allergy Unit, National Network ARADyAL, IBIMA-Regional University Hospital of Malaga-UMA (Pavilion C), Plaza del Hospital Civil, 29009 Malaga, Spain; 2 BIONAND-Andalusian Centre for Nanomedicine and Biotechnology, Malaga, Spain; 3 Allergy Unit, Presidio Columbus, Rome, Italy; 4 IRCCS Oasi Maria S.S., Troina, Italy; 5 Department of Chest Diseases, Division of Immunology and Allergy, Ankara University School of Medicine, Ankara, Turkey; 6 Hôpital Arnaud de Villeneuve, University Hospital of Montpellier and Sorbonne Universités, UPMC Paris 06, UMR-S 1136, IPLESP, Equipe EPAR, 75013 Paris, France; 7 Department of Internal Medicine, Division of Allergy and Immunology, University of Texas Southwestern Medical Center, Dallas, TX USA; 8 Kaiser Permanente Health Care Program, San Diego, CA USA; 9 Mayo Clinic College of Medicine, Division of Allergic Diseases, Mayo Clinic, Rochester, MN 55905 USA; 10 Division of Rheumatology, Allergy and Immunology, Department of Medicine, Massachusetts General Hospital and Harvard Medical School, Boston, MA USA; 11 Department of Dermatology, Medical University of Graz, Graz, Austria; 12 Division of Rheumatology, Immunology and Allergy, Brigham and Women’s Hospital, Boston, MSA USA; 13 Sorbonne Universities, UPMC Univ Paris 06, Dermatology and Allergology Department, Tenon Hospital (AP-HP), 4 rue de la chine, 75020 Paris, France; 14 Allergy Unit, IBIMA-Regional University Hospital of Malaga-UMA (Pavilion C), Plaza del Hospital Civil, 29009 Malaga, Spain; 15 Allergy Unit, Azienda Ospedaliera Universitaria Intergrata of Verona, Verona, Italy

**Keywords:** Drug, Hypersensitivity, Allergy, Diagnosis, Skin test, In vitro test, IgE, T-cells, Drug provocation test, Sensitization, United States, Europe

## Abstract

Drug hypersensitivity reactions (DHRs) affect an unknown proportion of the general population, and are an important public health problem due to their potential to cause life-threatening anaphylaxis and rare severe cutaneous allergic reactions. DHR evaluations are frequently needed in both ambulatory and hospital settings and have a complex diagnosis that requires a detailed clinical history and other tests that may include in vitro tests and in vivo procedures such as skin tests and drug provocation tests. Although over the years both European and U.S. experts have published statements on general procedures for evaluating DHRs, a substantial discordance in their daily management exists. In this review, we highlight both the differences and the similarities between the European and U.S. perspectives. While a general consensus exists on the importance of skin tests for evaluating DHRs, concordance between Americans and Europeans exists solely regarding their use in immediate reactions and the fact that a confirmation of a presumptive diagnosis by drug provocation tests is often the only reliable way to establish a diagnosis. Finally, great heterogeneity exists in the application of in vitro tests, which require further study to be well validated.

## Background

Drugs can induce immunologically mediated adverse reactions that, together with non-allergic direct mast-cell mediated drug hypersensitivity reactions (DHRs), comprise 15% of all adverse drug reactions [1]. Only when a definite immunological mechanism (either clinically significant drug-specific IgE or IgG antibodies or T cells) is demonstrated, these reactions should be classified as drug allergic reactions [2].

DHRs are commonly classified as immediate (IDHR) or non-immediate (NIDHR) depending on their onset during treatment [3]. IDHRs are mainly induced by an IgE- or IgG and complement-mediated mechanism and occur within 1–6 h after the last drug administration. NIDHRs occur at least 1 h after the initial drug administration in sensitized patients, but usually after several hours or even days, and are often associated with a delayed IgG-mediated or T cell-dependent mechanism [4].

While clinically IDHRs can affect any organ system, the skin is the most frequently involved, with the most common manifestation being urticaria/angioedema and the most severe being anaphylaxis/anaphylactic shock. The most common clinical manifestations of NIDHR are benign rashes, especially urticaria and maculopapular eruptions. However, serious cutaneous adverse reactions (SCAR)—such as, acute generalized exanthematous pustulosis (AGEP), toxic epidermal necrolysis (TEN), Stevens–Johnson syndrome (SJS), and drug reaction with eosinophilia and systemic symptoms (DRESS) or drug-induced hypersensitivity syndrome (DiHS)—can rarely occur [4].

Drug intolerances are reported in the medical records of about 8% of the populations that use modern healthcare, but only a small minority of them are indisputably immunologically mediated DHRs. Though DHRs affect an unknown proportion of the general population, they are an important public health problem because of their potential to cause life-threatening anaphylaxis and rare SCAR. Drug intolerance reports dramatically alter medications individuals are subsequently exposed to and can cause additional morbidity because of the consequences of sub-optimal pharmacotherapy. DHR evaluations are a frequent demand in both ambulatory and hospital settings. DHRs can pose complex diagnostic problems requiring a detailed clinical history and other tests that may include in vitro tests and in vivo procedures, such as skin tests (STs) and drug provocation tests (DPTs), also called graded challenges or test doses in the U.S. literature.

The reference standard for drug tolerance is no evidence of reaction after a therapeutic dose of the implicated drug. For a suspected IgE-mediated IDHR at least 1–6 h of observation are necessary. For a suspected T-cell mediated NIDHR 5 days-several weeks of observation may be necessary. Diagnostic testing, which may include STs and in vitro testing, is performed to minimize the number of serious positive DPTs. Over the years both European and U.S. experts have published statements on general procedures for evaluating DHRs [5–7]. However, there are still substantial differences in the daily management of DHRs around the world.

In this review, the similarities and differences between European and U.S. perspectives of management of DHRs will be discussed.

## Skin tests

In both the European [6, 8] and American [9] guidelines, STs are considered of paramount importance for evaluating DHRs. The European guidelines (EUgd) [6, 8] advise applying STs according to the suspected pathogenic mechanism of the DHRs. In IDHRs to β-lactams, for example, an IgE-mediated pathogenic mechanism can be demonstrated by a positive skin prick test (SPT) and/or intradermal test (IDT) after 20-min, whereas in NIDHRs, often a positive patch test (PT) and/or a late-reading IDT is found after several hours or days, indicating a T-cell-mediated pathogenic mechanism.

The U.S. practice parameter (USpp) [9] highlights mainly the usefulness of STs for assessing IDHRs to drugs, such as benzylpenicillin, insulin, heterologous antisera, and streptokinase, for which optimal negative predictive values (NPV) for IgE-mediated reactions have been established. According to this parameter [9], a positive immediate-reading ST result using a non-irritating concentration of a drug suggests that the patient has drug-specific IgE (sIgE) and may be at significant risk for anaphylaxis or less severe IDHRs.

The manner in which NIDHRs are evaluated differs significantly between the U.S. and Europe [10]. In European centres, patients with NIDHRs are evaluated by both PTs and delayed-reading IDTs [8], including those with severe NIDHRs, such as TEN/SJS, DRESS/DiHS, and AGEP [8, 11, 12]. PTs are usually performed first (i.e., prior to IDTs) and if positive, IDTs are avoided; if PTs are negative, in subjects with severe NIDHRs IDTs are performed using higher drug dilutions [8]. The clinical relevance of PTs or IDTs is not clear as, given the severity of these reactions, DPTs to confirm true allergy are not done.

In the U.S., PTs and delayed-reading IDTs are not routinely performed, probably because of limited data on test sensitivity combined with U.S. practice differences. For example, patients with SCAR may be diagnosed and managed by dermatologists who do not perform PTs or STs, with allergists only infrequently managing NIDHRs (i.e., when the clinical history and biopsy are not conclusive). For benign maculopapular rashes and fixed drug eruption (FDE), a positive PT or IDT would not change management recommendations, and would therefore probably not be incorporated into U.S. clinical practice. The USpp on contact dermatitis [13] states that PT to drugs may have a role in some NIDHRs, such as maculopapular rashes, AGEP, and FDE. This parameter gives a weak recommendation for PTs based on low quality evidence stating that: “there is no standardized approach to define the population, clinical manifestation, drug to PT, and PT materials to make PTs to drugs a standard of care”.

Recently, the European Network on Drug Allergy (ENDA) and European Academy of Allergy and Clinical Immunology (EAACI) Interest Group on Drug Allergy wrote a position paper on ST concentrations for systematically administered drugs [6]. However, it was possible to recommend specific drug concentrations only for β-lactam antibiotics, perioperative drugs, heparins, platinum salts, and iodinated contrast media (ICM). In effect, ST sensitivity appeared to be moderate to high for immediate reactions to these drugs, but low for many other drugs.

### β-Lactam antibiotics

#### Immediate reactions

In both the EUgd [14, 15] and USpp [9], STs represent the first-line method for diagnosing IDHRs to β-lactams (Table 1). With regard to benzylpenicillin [9, 14, 15], STs should be performed with the classic benzylpenicillin reagents: benzylpenicilloyl-poly-l-lysine (PPL), minor determinant mixture (MDM: benzylpenicillin, benzylpenilloate and benzylpenicilloate), and benzylpenicillin itself. In the US, the only minor determinant commercially available is benzylpenicillin and thus most US allergists do not routinely test with a complete MDM. The highest concentrations recommended in both SPTs and IDTs and the commercially available kits of penicillin reagents are shown in Table 1. It should be noted that, in the aforesaid European documents [6, 14, 15], the correct values of reagent concentrations expressed in mol/L were changed incorrectly to mmol/L.

**Table 1:** Similarities and differences between Americans and Europeans in the management of β-lactam antibiotic hypersensitivity

	American perspective	European perspective	Comment
General rules	Evaluation mainly by signs and symptoms In history of severe reactions^a^: avoidance of allergy tests In history of non-severe reactions: diagnostic approach can be applied	Evaluation by clinical history and allergy tests	Different
DPTs	Recommended if other diagnostic tools are negative Consider contraindications	Recommended if other diagnostic tools are negative Consider contraindications	Similar
Desensitization	Recommended Consider indications and contraindications	Recommended Consider indications and contraindications	Similar
*Immediate reactions*			
General rules	SPTs followed by IDTs are the first to perform Perform DPTs if STs are negative	SPTs followed by IDTs are the first to perform Perform DPTs if STs and in vitro tests are negative	Similar
STs	*Penicillins*		
	Recommended: PPL: 5 × 10^−5^ mol/L MDM: 2 × 10^−2^ mol/L BP: 10,000 IU/mL	Recommended PPL: 5 × 10^−5^ mol/L MDM: 2 × 10^−2^ mol/L BP: 10,000 IU/mL	Similar
	*Semisynthetic penicillins*		
	Not routinely recommended^a^	Recommended AX: 20 mg/mL AMP: 20 mg/mL	Different
	*β-Lactamase inhibitors*		
	Not recommended^a^	Recommended with original drug and the individual components of the antibiotic combination	Different
	*Cephalosporins*		
	Not recommended^a^	Recommended with original drug (max: 2–20 mg/mL)	Different
	*Aztreonam/carbapenems*		
	Not recommended^a^	Recommended Aztreonam: 2 mg/mL; Imipenem/cilastatin: 0.5 mg/mL of each component; Meropenem: 1 mg/mL; Ertapenem: 1 mg/mL	Different
	*Commercially available kits*		
	PRE-PEN^®^ (AllerQuest LLC, Plainville, CT, USA) PPL: 6.0 × 10^−5^ mol/L	DAP^®^ (Diater, Leganés, Madrid, Spain) BP-OL: 0.04 mg/mL (8.64 × 10^−5^ mol/L) MD: Benzylpenilloate 0.5 mg/mL (1.5 × 10^−3^ mol/L) AX: 20 mg/mL	Different
In vitro tests	*Serum specific IgE assays*		
	Not recommended	Testing with penicillins is recommended	Different
	*Basophil activation tests*		
	Not recommended	Recommended as complementary to sIgE	Different
*Non-immediate reactions*			
STs/PTs	Not recommended	PTs followed by delayed-reading IDTs are recommended in routine approach In case of positive PTs, IDTs may be avoided	Different
In vitro tests	Not recommended	Not recommended	Similar
*Retest*			
	Repeating penicillin STs routinely is not indicated in patients with a history of non-severe penicillin reactions who have tolerated 1 or more oral penicillin courses	Weakly recommended: retesting (2–4 weeks later) patients who suffered severe immediate reactions to BLs and display negative results in the first allergy evaluation, including DPTs	

*DPTs* drug provocation tests, *STs* skin tests, *SPTs* skin prick tests, *IDTs* intradermal tests, *PTs* patch tests, *PPL* benzylpenicilloyl-poly-l-lysine, *POL* benzylpenicilloyl-octa-l-lysine, *MDM* minor determinant mixture, *MD* minor determinant, *BP* Benzylpenicillin, *AX* amoxicillin, *AMP* ampicillin, *sIgE* specific IgE

^a^Due to unknown negative predictive values of STs

According to the USpp [9], the NPV of STs with classic penicillin reagents approaches 100%, whereas the positive predictive value (PPV) is between 40 and 100%. STs with PPL and benzylpenicillin only (without benzylpenicilloate or benzylpenilloate) appear to have adequate NPV in the evaluation of benzylpenicillin allergy, but not amoxicillin or other β-lactam allergies. On the contrary, the NPV of STs without PPL is poor because many allergic patients show ST reactivity only to the major determinant.

As far as IDHRs to semisynthetic penicillins are concerned, in Europe, amoxicillin, ampicillin, and other suspected semisynthetic penicillins for parenteral administration are recommended for STs at concentrations up to 20 mg/mL [6], in addition to PPL, MDM, and benzylpenicillin. The final concentration of these penicillins, which are sodium salts, ranges from 100 to 200 mg/mL; thus it is easy to obtain a solution of 20 mg/mL. On the other hand, the USpp [9] states that: “The NPV of STs with native semisynthetic penicillins is unknown, and there is no consensus regarding the appropriate concentration that should be used”. In any case, in North America, while ampicillin is available, the trihydrate of amoxicillin has been used, which limits the concentration that can be prepared for STs to about 4 mg/mL [16, 17]. In the U.S., an amoxicillin challenge following negative benzylpenicillin STs is routinely done, eliminating the need for aminopenicillin STs given the rarity of side-chain specific reactions in the U.S.

When β-lactams are used in combination with a β-lactamase inhibitor (e.g., amoxicillin and clavulanic acid), the EUgd [6, 15] recommend STs with the original drug and individual components of the antibiotic combination.

For the investigation of IDHRs to cephalosporins, according to the EUgd [6, 14, 15], the suspected cephalosporin, PPL, MDM and β-lactams with similar side chains are used. The highest cephalosporin concentration recommended by the EUgd in both SPTs and IDTs is 2 mg/mL [6]. However, taking into account some studies [12, 18], the EUgd also state that: “… for cefuroxime, ceftriaxone, cefotaxime, ceftazidime, cefazolin, cephalexin, cefaclor, and cefatrizine, but not cefepime, concentrations up to 20 mg/mL are probably also not irritant and might improve the sensitivity without affecting the specificity” [18]. On the other hand, the USpp [9] states that: “STs with native cephalosporins is not standardized, but a positive ST result using a non-irritating concentration suggests the presence of drug specific IgE antibodies. A negative ST result does not rule out an allergy because the NPV is unknown”.

With regard to aztreonam and carbapenems, the USpp [9] states that STs with a non-irritating concentration of native antibiotics have the same limitation and questionable NPV as with cephalosporins.

#### Non-immediate reactions

The EUgd [15, 19] recommend assessing NIDHRs to β-lactams by both PTs and delayed-reading IDTs. PTs with the suspected β-lactams are usually performed first; if positive, IDTs may be avoided. Delayed-reading IDTs generally have a higher sensitivity than PTs, with a similar specificity [20], and STs with PPL and MDM are scarcely useful [20]. PTs and delayed-reading IDTs are negative in most patients who experienced mild NIDHRs, particularly to cephalosporins [21], and therefore, PTs may be avoided [22].

The most common approach in the U.S. is to diagnose NIDHRs to β-lactams based upon signs and symptoms [10]. In patients with severe reactions, β-lactams are simply avoided. If the NIDHRs involved hives or angioedema, the patient may be evaluated for IgE-mediated allergy to the β-lactam that caused the reaction, as well as the β-lactam that is needed in the immediate future. If the patient’s past reaction did not involve hives or angioedema and was mild (e.g., maculopapular rash), according to the USpp [9], penicillin STs should be considered before a future course of penicillin is given. In case of a negative result, DPTs to the desired β-lactam can be performed.

### Non-β-lactam antibiotics

Both the USpp [9] and EUgd [6] agree that STs with non-irritating concentrations of non-β-lactam antibiotics are not standardized. According to the latter document [6], for most non-β-lactam antibiotics, the value of STs appears to be uncertain and false-positive reactions may occur when the antibiotic is tested at high concentrations. The EUgd [6, 8] recommend studying NIDHRs to non-β-lactam antibiotics by using PTs and delayed-reading IDTs.

### Non-steroidal anti-inflammatory drugs (NSAIDs)

The USpp [9] and EUgd [6] agree that most IDHRs to NSAIDs (excluding pyrazolones) are not IgE-mediated but related to an aberrant arachidonic acid metabolism. Therefore, the EUgd recommend performing STs with pyrazolones although sensitivity is not optimal and risk of systemic responses after IDTs exists [23]. In the U.S., pyrazolones are not available and NSAID STs are not recommended and rarely performed.

Considering non-pyrazolone single-NSAID-induced urticaria/angioedema or anaphylaxis (SNIUAA), STs with the culprit drug may be performed to confirm a selective, IgE-mediated type of hypersensitivity, although their usefulness has not been proven in large series [23]. In this regard, the EUgd [6] indicate that the irritating potential of all NSAIDs appears to be low in SPTs, and the specificity is thus high (>95%); for IDTs, up to 0.1 mg/mL appear to not irritate the skin.

In NIDHRs [6, 23], PTs and delayed-reading IDTs show low sensitivity, but high specificity. Delayed-reading IDTs with NSAIDs, particularly metamizol, are more sensitive than PTs. The latter tests with up to 10% or even 30% NSAID in petrolatum do not seem to irritate the skin, “although the additional value of using the higher concentration is questionable” [6].

### Cancer chemotherapeutic agents

According to both the USpp [9] and EUgd [6], STs are useful for evaluating platinum salt-related IDHRs, while for other chemotherapeutic drugs (i.e., taxol) experience is limited and clinical usefulness not clear. For platinum salts (cisplatin, carboplatin, and oxaliplatin), the use of undiluted drugs is recommended by the American document [9] for diagnosing hypersensitivity, identifying patients at risk, and determining the indication and protocol of desensitization [24].

The EUgd [6] state that the irritant potential of chemotherapeutic drugs is low and recommend SPTs with carboplatin at 10 mg/mL, oxaliplatin at 1 mg/mL, and cisplatin at 1 mg/mL, and IDTs at 1, 0.1 and 0.1 mg/mL, respectively. For other chemotherapeutic drugs, SPTs with undiluted agents are probably non-irritant, but due to toxicity concerns, a general recommendation cannot be given [6] and for IDTs, a 1/10 dilution may be used. IDTs with carboplatin at 10 mg/mL can cause skin necrosis and scarring and should be avoided [25]. PTs are almost always negative; thus, they are not recommended [6].

### Perioperative agents

According to both the USpp [9] and EUgd [6], the evaluation of hypersensitivity reactions to perioperative agents should include STs with all substances the patient was exposed to, including antibiotics, colloids, latex, disinfectants, opioids, blue dyes, etc. Recommended ST concentrations are shown in Table 2 [6, 26]. Both the American [9] and European documents [6] agree that neuromuscular blocking agents (NMBAs) and opiates can induce non-specific histamine release in the skin, increasing the possibility of false-positive tests, especially IDTs. The latter document [6] recommends performing IDTs with a panel of NMBAs, including the suspected one, in order to assess cross-reactivity and identify safe alternatives, and not carrying out routinely preoperative screening in patients without prior reactions.

**Table 2:** Skin test concentrations for perioperative agents

	ENDA proposal		American proposal	
	SPT (mg/mL)	IDT (mg/mL)	SPT (mg/mL)	IDT (mg/mL)
Thiopental	25	2.5		0.2
Propofol	10	1	10–1	10–0.1
Ketamine	10	1	10	0.25
Etomidate	2	0.2	2	0.2–0.002
Midazolam	5	0.5	5	0.5–0.25
Fentanyl	0.05	0.005	0.05	0.005–0.000005
Alfentanil	0.5	0.05		
Sufentanil	0.005	0.0005		
Remifentanil	0.05	0.005		
Morphine	1	0.01		
Atracurium	1	0.01	10	0.01
Cis-atracurium	2	0.02	2	0.01–0.001
Mivacurium	0.2	0.002		
Rocuronium	10	0.05	10	0.01–0.001
Vecuronium	4	0.4	10	0.1–0.001
Pancuronium	2	0.2	2	0.02
Suxamethonium	10	0.1		
Chlorhexidine	5	0.002	1.2	0.00048
Alcuronium				0.005
Methohexital				0.1
Metocurine				0.002
Succinylcholine			20	0.02–0.05
Thioamyl				0.1
Tubocuranine				0.0003–0.0001

*ENDA* European Network for Drug Allergy, *SPTs* skin prick tests, *IDTs* Intradermal tests

The USpp on drug allergy [9] and the ones on the diagnosis and management of anaphylaxis [26, 27] highlight the usefulness of STs with thiopental, protamine, propofol, and blue dyes (e.g., methylene blue, isosulfan blue, and patent blue V). Moreover, chlorhexidine is an integral part of the perioperative test panel in some European and U.S. centres [28, 29].

Finally, there are no recommendations for evaluating NIDHRs to perioperative drugs [6] except opiates, although there is no universal agreement on the optimal vehicle (aqua, petrolatum, ethanol) or test concentration.

### Local anaesthetics

The USpp [9] and EUgd [6] agree that hypersensitivity reactions to local anaesthetics (LAs) are rare. According to the USpp [9], a SPT with the undiluted anaesthetic and a DPT is the reasonable approach. The EUgd [6] recommend using neat LAs for SPTs and a 1/10 dilution for IDTs. As cross-reactivity has been reported among ester-type LAs, but not among amide LAs, in confirmed LA allergy, this document [6] also recommends testing other LAs in order to identify a safe alternative. For NIDHRs, it [6] recommends IDT with a 1/10 dilution of LAs and PTs with neat LAs. The USpp on contact dermatitis indicates PTs for NIDHRs [13].

### Iodinated contrast media, gadolinium chelates and dyes

The USpp [9] does not consider STs a useful tool in evaluating hypersensitivity reactions to ICM, arguing that such reactions are non-IgE mediated with rare exceptions. Moreover, although it states that the mechanism of NIDHRs to ICM appears to be T-cell mediated, as happens with other drugs in the U.S., both PTs and delayed-reading STs are not indicated [9]. On the contrary, the EUgd recommend STs with a panel of ICM to diagnose cross-reactivity and identify safe alternatives. In NIDHRs, both delayed-reading IDTs and PTs should be carried out to enhance sensitivity; however, false-negative STs may occur [6]. According to this document [6], SPTs with ICM, gadolinium chelates, blue dyes (patent and methylene), and fluorescein are performed undiluted, whereas IDTs with drugs 1/10 diluted (except for methylene blue, which is 1/100). PTs are only applied with ICM and fluorescein undiluted.

### Other drugs

The USpp [9] and EUgd [6] agree that STs are useful for evaluating DHRs, such as those to insulin, corticosteroids, and heparins. The EUgd [6] highlight the importance of evaluating NIDHRs to these drugs by using PTs and delayed-reading IDTs and provide information on non-irritant concentrations for STs with several other drugs, such as biological agents (e.g., adalimumab, etanercept, infliximab, and omalizumab), proton pump inhibitors, H_2_ antihistamines, antihypertensive drugs (i.e., calcium channel blockers and β-blockers), vaccines, abacavir, and anticonvulsants.

## In vitro tests

The selection of in vitro methods mainly depends on the mechanisms involved, IgE- or T-cell-mediated, and their availability for a specific drug. Although they have great advantages, their value in real life conditions is not clear due to the lack of well-controlled studies with a sufficient number of confirmed cases. Moreover, evident differences exist between the American and European points of view [4, 30, 31]. The recent ENDA position paper [30] concluded that, although many in vitro tests could help in diagnosis, few showed at least grade B recommendation (Table 3). The National Institute of Allergy and Infectious Diseases (NIAID) Division of Allergy, Immunology and Transplantation [31] recommends in vitro tests for diagnosing IDHRs when an IgE mechanism is likely but STs are neither available nor validated. In an attempt to include European and American organizations, the ICON (International CONsensus) document [4] indicated the need for new and validated biological diagnostic tests, available to all clinicians, in order to improve care for these patients.

**Table 3:** In vitro tests to identify culprit drugs based on underlying immunological mechanism

	Effector cells	Test	Detection	Drug studied	Limitations
Immediate reactions	Mast cells and basophils	Immunoassay	Serum tryptase (normal values: <11.4 ng/mL)	Any drug involved in grade 2–3 anaphylactic reactions	Samples must be obtained within 60–90 min after the reaction
			Specific IgE	β-Lactams, NMBAs, chlorhexidine, and biological agents	Available for few drugs Low sensitivity
		Basophil activation test	Activated basophils	β-Lactams, NMBAs, pyrazolones, and fluoroquinolones Potentially available for any injectable drug	Need for fresh blood Low sensitivity
Non-immediate reactions	Cell-mediated: T-cells, NK cells, neutrophils, monocytes	HLA-allele determination	Allele determination	Abacavir, carbamazepine	Associations are very specific for particular drugs and/or types of DHRs Variable sensitivity
		Lymphocyte transformation test	Lymphocyte proliferation	Potentially available for any injectable drug	Limited supportive data Low sensitivity/specificity
		ELISpot	Number of cells producing inflammatory markers	Potentially available for any injectable drug	Limited supportive data Low sensitivity/specificity

*NMBAs* neuromuscular blocking agents, *DHRs* drug hypersensitivity reactions, *ELISpot* enzyme-linked immunosorbent spot

### Tryptase level

The serum tryptase level can be helpful to confirm a diagnosis of anaphylaxis in IDHRs, especially if blood is drawn 60–90 min after symptom onset, with on-going symptoms, and at least 24 h after resolution. The USpp recommends tryptase evaluation in all patients with known or suspected anaphylaxis [9].

### Specific IgE determination

The detection of drug-sIgE in serum is based on immunoassays: radioimmunoassay, enzymoimmunoassay, and fluoroimmunoassay (FEIA). The most widely used commercial method is the FEIA (ImmunoCAP, Thermofisher, Uppsala, Sweden), although it is only suitable for a limited number of drugs, including some β-lactams, NMBAs, chlorhexidine, and biological agents. Its sensitivity depends on the drug involved, being generally rather low (0–50%) for β-lactams [32–38], variable (44–92%) for NMBAs [39–41], and high for cetuximab (68–92%) and chlorhexidine (91.6%) [42–44]. Moreover, this test has shown to be concordant and complementary with STs [32, 33, 36–41, 44], although with lower sensitivity [9].

The EUgd recommend ImmunoCAP for diagnosing IDHRs to β-lactams, NMBAs, chlorhexidine, and biological agents; in case of severe reactions, it should be performed before STs [30]. In the U.S. experience, penicillin is the only low-molecular-weight agent for which validated testing has been documented [9], although with no concordance with STs and DPTs, indicating no apparent usefulness in diagnosing patients with histories of penicillin allergy [45]. A lack of specificity has been also found in a European report for penicillin V-sIgE [46].

Moreover, it is accepted in both European and North American guidelines that, although a positive penicillin in vitro test in the context of a suggestive history is highly predictive of an IgE-mediated allergy, a negative test does not rule out an IgE-mediated allergy [9, 30].

### Basophil activation test

There is an increasing interest in the basophil activation test (BAT), mainly because it can be used for many drugs. This test is based on the flow-cytometric determination of basophil activation after stimulation with the drugs or their metabolites. Although commercially available, protocols are not standardized between labs.

In Europe, there are validated studies for β-lactams, NMBA, pyrazolones, and fluoroquinolones, showing complementarity to STs. For β-lactams, including clavulanic acid, the sensitivity ranges from 22 to 55%, with a specificity of 79–96% [35, 47–50]. Regarding NMBAs, BAT sensitivity ranges from 64 to 85.7% and specificity from 93 to 100%, being especially high for rocuronium (91.7%) [41, 51, 52]. The BAT is useful for pyrazolones and fluoroquinolones, with a sensitivity of 42–55 [53, 54] and 36–71% [55, 56] respectively and specificity of 86–100%. Moreover, for fluoroquinolones, BAT has demonstrated a high NPV, useful for deciding whether to perform DPTs [55, 57].

Thus, the EUgd recommend the BAT in high-risk patients, when available, and before DPTs and even STs [30]. However, the USpp indicates that further confirmatory studies are needed since no commercially available BAT assay in the U.S. has proven validity [9]. Moreover, as with immunoassays, BAT should be performed within 1 year after healing [54, 58, 59].

More controversy exists about the role of BAT in non-allergic hypersensitivity to NSAIDs [23] with a great variability in sensitivity and specificity among studies [60–63]. Importantly, basophil activation by NSAIDs occurs to a variable extent in healthy individuals who tolerate NSAIDs, decreasing test specificity and NPV. All these data have generated a general consensus between European and North American guidelines, indicating that BAT is not useful for diagnosing non-allergic hypersensitivity to NSAIDs [51, 61–64].

### HLA-allele determination

Different studies have indicated strong associations of some HLA alleles with a high risk of severe T-cell mediated reactions to drugs like abacavir, carbamazepine, and allopurinol. HLAB*57:01 is associated with DRESS/DiHS induced by abacavir in most ethnic populations (sensitivity: 45.5–80%; specificity: 97.6–99%) [65–67]. Moreover, HLA-B*57:01 screening reduced the prevalence of abacavir-induced DHRs from 7.8% in controls to 3.4% in screened patients, demonstrating that this genetic testing is cost-effective in many countries [65]; however, it should be noted that this screening does not prevent other types of abacavir DHRs [31].

For carbamazepine, HLA-B*15:02 has been strongly associated with SJS/TEN in Asian populations [68–71], thus the European Medicines Agency and U.S. Food and Drug Administration recommend its screening before starting a treatment with this drug in at-risk populations [72]. HLA-B*58:01 allele is associated with a high risk of allopurinol-induced DRESS and SJS/TEN in Asian and Caucasian populations [73, 74] and its screening is recommended by the American College of Rheumatology but not by the U.S. Food and Drug Administration. Given the low prevalence of allopurinol hypersensitivity and high prevalence of HLA-B*58:01 in Asian patients, the usefulness of screening has been questioned [72, 75]. Similar recommendations are made in a recent ENDA review article [30].

With these data there is a consensus about the need of performing genetic testing for specific drugs, although their predictive values need to be improved [31].

### Lymphocyte transformation test

This test determines the proliferation of drug specific T-cells upon stimulation with suspected drug(s). The sensitivity and specificity are highly variable, and depend on the culprit drug [51, 76]. For β-lactams and anticonvulsants the lymphocyte transformation test (LTT) has demonstrated fair sensitivity (60–70%) and specificity (85–93%) [77]. LTT sensitivity also depends on the type of reaction, being quite high in MPE, FDE, AGEP, and DRESS, but low in SJS/TEN [51].

The European and North American guidelines [9, 30] indicate that although the LTT may be useful as a retrospective indicator of cell-mediated DHRs, its PPV and NPV have not been determined and it is not available in most centres. Thus, to increase its clinical applicability, large-scale studies are needed.

### Enzyme-linked immunosorbent spot

Enzyme-linked immunosorbent spot (ELISpot) measures cells secreting different mediators upon drug stimulation and is useful for evaluating the specific effector response. Although recent studies its usefulness, especially in severe cases, neither American nor European guidelines currently recommend this testing [30, 78–80].

## Drug provocation tests

Drug allergy experts worldwide consider the DPT as the gold standard for the identification of a culprit drug in patients with a suspected DHR. However, the U.S. uses different terminology for DPTs, specifically graded challenges and test doses [9]. Both the European position paper [7] and the USpp [9] consider that DPTs are mainly intended to exclude a hypersensitivity in non-suggestive histories or to provide safe alternatives in allergic patients and thus prove tolerance. This is especially true since for many drugs there are no standardized STs [6] or in vitro tests [30] nor well established NPV and especially PPV of these tests. Only the European document [7] emphasizes their role in the establishment of a firm diagnosis of DHRs.

Similarities and differences have been previously highlighted in the ICON [4]. There are indeed many similarities between Europe and the U.S.; however, the biggest difference is in the clinical indication of when to undertake DPTs. The USpp [9] recommends DPTs only if the probability of DHRs is low and the clinical scenario justifies the possible risk, e.g., there is no comparable alternative medication. DPTs are also performed for patients with multiple drug allergy syndrome whose medical care is impacted by their challenging allergy list. It states that the “objective of a graded challenge is to introduce a medication cautiously so as not to induce a severe reaction” [9]. An exception described in the USpp [9] is the possible role of DPTs with aspirin in NSAID-exacerbated respiratory disease (NERD). Since there is no ST or in vitro test for NERD, where aspirin desensitization is very effective, if a definitive diagnosis is required, DPT is indicated. However, most U.S. drug allergy specialists desensitize in most cases if there is a highly suspected drug. DPT protocols vary and guidelines are only suggestive, not coercive. The USpp [9] considers that utilizing more than 4 or 5 steps may induce tolerance, whereas European DPT protocols often use 4–6 steps.

β-Lactam challenge protocols for NIDHRs vary considerably in the U.S. in terms of initial dosing (e.g., graded vs. full dose) and duration of challenge (1 day vs. extended several day challenges). In the U.S., Antibiotic Stewardship recommends minimizing antibiotic use [12]. Thus, most U.S. allergists no longer prescribe multiple day provocative challenges to antibiotics in patients who do not require antibiotic therapy.

The USpp [9] and European position paper [7] attribute different values to negative DPTs (Table 5). This divergence may be due to the different DPT aims (Europe: both to exclude and confirm diagnosis of DHRs vs. U.S.: mainly to exclude diagnosis of DHRs i.e., low clinical suspicion of DHR).

Both documents [7, 9] agree on the precautions, contraindications, and surveillance required for DPTs (Table 4), although they are generally safe procedures [81]. Finally, European and U.S. documents [9, 14, 15] mention potential resensitization by DPTs in subjects with hypersensitivity reactions to β-lactams (Table 5).

**Table 4:** Precautions and contraindications of performing drug provocation tests (DPTs) (from ICON with permission [4])

DPTs are contraindicated in non-controllable and/or severe life-threatening DHRs	Severe cutaneous reactions, such as SJS, TEN, DRESS, vasculitis, AGEP Systemic reactions such as DRESS, any internal organ involvement, hematological reactions Anaphylaxis may be tested after discussion with patient of risk and benefits
DPTs are not indicated	The offending drug is unlikely to be needed and several structurally unrelated alternatives exist Severe concurrent illness or pregnancy (unless the drug is essential for the concurrent illness or required during pregnancy or delivery)
DPTs should be performed under the highest safety conditions	Trained staff that are: familiar with allergy tests, can identify early signs of a positive reaction, and can manage life-threatening allergic reactions Emergency resuscitative equipment should be available

*DHRs* drug hypersensitivity reactions, *SJS* Stevens–Johnson syndrome, *TEN* toxic epidermal necrolysis, *DRESS* drug reaction with eosinophilia and systemic symptoms, *AGEP* acute generalized exanthematous pustulosis

**Table 5:** Similarities and differences between Americans and Europeans in the management of drug hypersensitivity

	American perspective	European perspective
General rules for management	Based on clinical evaluation For immediate reactions, STs are applied first; if they are negative, DPT is performed unless contraindication exists STs are not recommended for non-immediate reactions No allergy tests are recommended in history of severe reactions* Desensitization is recommended in indicated cases	Allergy tests are strongly recommended if there are no contraindications. STs are applied first; if they are negative, DPT is performed unless contraindication exists Selection of STs depends on underlying mechanisms (SPTs/IDTs for immediate reactions, PTs and delayed-reading IDTs for non-immediate reactions) Desensitization is recommended in indicated cases
*Immediate reactions*		
SPTs/IDTs	Recommended for BLs, other antibiotics, NMBAs, chlorhexidine, chemotherapeutic agents, insulin, heterologous antisera, and streptokinase at non-irritating concentrations	Recommended based on sensitivity and specificity of the tests BLs, pyrazolones, NMBAs, chlorhexidine, LAs, RCM (strong recommendation) Should be performed at non-irritating concentrations
In vitro tests	Not recommended	Serum specific IgE assays are recommended for BLs, NMBAs, and chlorhexidine BAT is recommended for BLs and NMBAs (complementary to sIgE assays), as well as for pyrazolones, fluoroquinolones, and RCM
*Non-immediate reactions*		
General rules for management	PTs and IDTs are not recommended routinely	PTs and IDTs are recommended routinely PTs are performed first; if negative, delayed-reading IDTs are performed if no contraindications exist
PTs	May have a role in delayed DHRs, such as MPE, AGEP, and FDE	Recommended In severe cases, lower drug concentrations are recommended
Delayed-reading IDTs	Not recommended	In severe cases, such as DRESS, AGEP, and TEN/SJS, can be performed after negative PTs and at higher drug dilutions
In vitro tests	Pre-screening with certain HLA alleles before introduction of abacavir and carbamazepine Other tests (e.g., LTT and ELISpot) are not recommended	Pre-screening with certain HLA alleles before introduction of abacavir and carbamazepine Other tests (e.g., LTT and ELISpot) arenot recommended
*Non allergic drug hypersensitivity*		
Skin tests	Not recommended	Not recommended
In vitro tests	Not recommended	Not recommended
*Drug provocation tests/challenges*		
Indication	To exclude DHR in non-suggestive histories or provide safe alternatives	To exclude DHR in non-suggestive histories or provide safe alternatives To confirm diagnosis
Methods	Similar contraindications and precautions	Similar contraindications and precautions
Comment on a negative test result	“Patients who tolerate a graded challenge are considered to not be allergic to the drug and are not at increased risk for future reactions compared with the general population”	“A negative test does not prove tolerance to the drug in the future, but rather that there is no DHR at the time of the challenge and to the doses challenged”

*DPTs* drug provocation tests, *STs* skin tests, *SPTs* skin prick tests, *IDTs* intradermal tests, *PTs* patch tests, *BP* benzylpenicillin, *NMBAs* neuromuscular blocking agents, *LAs* local anaesthetics, *RCM* radio contrast media, *BLs* β-lactams, *BAT* basophil activation test, *DHRs* drug hypersensitivity reactions, *MPE* maculopapular exanthema, *DRESS* drug reaction with eosinophilia and systemic symptoms, *SJS/TEN* Stevens–Johnson syndrome/toxic epidermal necrolysis, *AGEP* acute generalized exanthematous pustulosis, *FDE* fixed drug eruption, *sIgE* specific IgE, *LTT* lymphocyte transformation test, *ELISpot* enzyme-linked immunosorbent spot

^a^DRESS, TEN/SJS; AGEP, severe anaphylactic shock within the last year

## Conclusions

While a general consensus exists on the importance of STs for evaluating DHRs, Americans and Europeans agree solely on their use for IDHRs (Table 5). Although in vitro tests may be helpful, particularly for severe life-threatening DHRs when STs are negative or not possible or contraindicated, they are used almost exclusively in Europe. In order to improve drug allergy testing, we must standardize protocols, and perform large, multi-site studies in well-characterized patients diagnosed by STs and/or DPTs when possible, to confirm drug-specific sensitivity, specificity, NPV, and PPV. Finally, DPTs are often the only reliable way to establish a diagnosis, but this procedure should be undertaken only in case of a compelling need and with caution. Regarding these matters, the position papers from both continents completely agree.

## Abbreviations

DHR: drug hypersensitivity reaction; IDHR: immediate drug hypersensitivity reaction; NIDHR: non-Immediate drug hypersensitivity reaction; SCAR: serious cutaneous adverse reaction; AGEP: acute generalized exanthematous pustulosis; TEN: toxic epidermal necrolysis; SJS: Stevens–Johnson syndrome; DRESS: drug reaction with eosinophilia and systemic symptoms; DiHS: drug-induced hypersensitivity syndrome; FDE: fixed drug eruption; ST: skin test; DPT: drug provocation test; EUgd: European guidelines; SPT: skin prick test; IDT: intradermal test; PT: patch test; USpp: US practice parameter; NPV: negative predictive value; sIgE: specific IgE; ENDA: European Network on Drug Allergy; EAACI: European Academy of Allergy and Clinical Immunology; ICM: iodinated contrast media; PPL: benzylpencilloyl-poly-l-lysine; MDM: minor determinant mixture; PPV: positive predictive value; NSAID: non-steroidal anti-inflammatory drug; SNIUAA: single-NSAID-induced urticaria/angioedema or anaphylaxis; NMBA: neuromuscular blocking agent; LA: local anaesthetic; NIAID: National Institute of Allergy and Infectious Diseases; ICON: International CONsensus; BAT: basophil activation test; LTT: lymphocyte transformation test; ELISpot: enzyme-linked immunosorbent spot; FEIA: fluoroimmunoassay; NERD: NSAID-exacerbated respiratory disease.

## References

[1] Gomes ER, Demoly P (2005). Epidemiology of hypersensitivity drug reactions. Curr Opin Allergy Clin Immunol.

[2] Johansson SG, Bieber T, Dahl R, Friedmann PS, Lanier BQ, Lockey RF (2004). Revised nomenclature for allergy for global use: Report of the Nomenclature Review Committee of the World Allergy Organization, October 2003. J Allergy Clin Immunol.

[3] Bircher AJ, Scherer Hofmeier K (2012). Drug hypersensitivity reactions: inconsistency in the use of the classification of immediate and nonimmediate reactions. J Allergy Clin Immunol..

[4] Demoly P, Adkinson NF, Brockow K, Castells M, Chiriac AM, Greenberger PA (2014). International consensus on drug allergy. Allergy.

[5] Demoly P, Kropf R, Bircher A, Pichler WJ (1999). Drug hypersensitivity: questionnaire. EAACI interest group on drug hypersensitivity. Allergy.

[6] Brockow K, Garvey LH, Aberer W, Atanaskovic-Markovic M, Barbaud A, Bilo MB (2013). Skin test concentrations for systemically administered drugs—an ENDA/EAACI Drug Allergy Interest Group position paper. Allergy.

[7] Aberer W, Bircher A, Romano A, Blanca M, Campi P, Fernandez J (2003). Drug provocation testing in the diagnosis of drug hypersensitivity reactions: general considerations. Allergy.

[8] Brockow K, Romano A, Blanca M, Ring J, Pichler W, Demoly P (2002). General considerations for skin test procedures in the diagnosis of drug hypersensitivity. Allergy.

[9] Joint Task Force on Practice P, American Academy of Allergy A, Immunology, American College of Allergy A, Immunology, Joint Council of Allergy A, et al. Drug allergy: an updated practice parameter. Ann Allergy Asthma Immunol. 2010;105(4):259–273. 10.1016/j.anai.2010.08.002 20934625

[10] Romano A, Warrington R. Antibiotic allergy. Immunol Allergy Clin N Am. 2014;34(3):489–506, vii. 10.1016/j.iac.2014.03.003 25017674

[11] Barbaud A, Collet E, Milpied B, Assier H, Staumont D, Avenel-Audran M (2013). A multicentre study to determine the value and safety of drug patch tests for the three main classes of severe cutaneous adverse drug reactions. Br J Dermatol.

[12] Romano A, Gaeta F, Valluzzi RL, Maggioletti M, Caruso C, Quaratino D (2016). Cross-reactivity and tolerability of aztreonam and cephalosporins in subjects with a T cell-mediated hypersensitivity to penicillins. J Allergy Clin Immunol.

[13] Fonacier L, Bernstein DI, Pacheco K, Holness DL, Blessing-Moore J, Khan D (2015). Contact dermatitis: a practice parameter-update 2015. J Allergy Clin Immunol Pract.

[14] Torres MJ, Blanca M, Fernandez J, Romano A, Weck A, Aberer W (2003). Diagnosis of immediate allergic reactions to beta-lactam antibiotics. Allergy.

[15] Blanca M, Romano A, Torres MJ, Fernandez J, Mayorga C, Rodriguez J (2009). Update on the evaluation of hypersensitivity reactions to betalactams. Allergy.

[16] Macy E (2011). The clinical evaluation of penicillin allergy: what is necessary, sufficient and safe given the materials currently available?. Clin Exp Allergy.

[17] Montanez MI, Torres MJ, Perez-Inestrosa E, Blanca M (2011). Clarification concerning amoxicillin skin testing. J Allergy Clin Immunol.

[18] Testi S, Severino M, Iorno ML, Capretti S, Ermini G, Macchia D (2010). Nonirritating concentration for skin testing with cephalosporins. J Investig Allergol Clin Immunol.

[19] Romano A, Blanca M, Torres MJ, Bircher A, Aberer W, Brockow K (2004). Diagnosis of nonimmediate reactions to beta-lactam antibiotics. Allergy.

[20] Romano A, Gaeta F, Valluzzi RL, Caruso C, Rumi G, Bousquet PJ (2010). The very limited usefulness of skin testing with penicilloyl-polylysine and the minor determinant mixture in evaluating nonimmediate reactions to penicillins. Allergy.

[21] Romano A, Gaeta F, Valluzzi RL, Caruso C, Alonzi C, Viola M (2012). Diagnosing nonimmediate reactions to cephalosporins. J Allergy Clin Immunol.

[22] Romano A, Caubet JC (2014). Antibiotic allergies in children and adults: from clinical symptoms to skin testing diagnosis. J Allergy Clin Immunol Pract.

[23] Kowalski ML, Asero R, Bavbek S, Blanca M, Blanca-Lopez N, Bochenek G (2013). Classification and practical approach to the diagnosis and management of hypersensitivity to nonsteroidal anti-inflammatory drugs. Allergy.

[24] Wang AL, Patil SU, Long AA, Banerji A (2015). Risk-stratification protocol for carboplatin and oxaliplatin hypersensitivity: repeat skin testing to identify drug allergy. Ann Allergy Asthma Immunol.

[25] Patil SU, Long AA, Ling M, Wilson MT, Hesterberg P, Wong JT (2012). A protocol for risk stratification of patients with carboplatin-induced hypersensitivity reactions. J Allergy Clin Immunol.

[26] Lieberman P, Nicklas RA, Oppenheimer J, Kemp SF, Lang DM, Bernstein DI (2010). The diagnosis and management of anaphylaxis practice parameter: 2010 update. J Allergy Clin Immunol.

[27] Lieberman P, Nicklas RA, Randolph C, Oppenheimer J, Bernstein D, Bernstein J (2015). Anaphylaxis—a practice parameter update 2015. Ann Allergy Asthma Immunol.

[28] Opstrup MS, Malling HJ, Kroigaard M, Mosbech H, Skov PS, Poulsen LK (2014). Standardized testing with chlorhexidine in perioperative allergy—a large single-centre evaluation. Allergy.

[29] Kuhlen JL, Camargo CA, Balekian DS, Blumenthal KG, Guyer A, Morris T (2016). Antibiotics are the most commonly identified cause of perioperative hypersensitivity reactions. J Allergy Clin Immunol Pract.

[30] Mayorga C, Celik G, Rouzaire P, Whitaker P, Bonadonna P, Cernadas JR, et al. In vitro tests for drug hypersensitivity reactions. An ENDA/EAACI drug allergy interest group position paper. Allergy. 2016;71:1103–34. 10.1111/all.12886 26991315

[31] Wheatley LM, Plaut M, Schwaninger JM, Banerji A, Castells M, Finkelman FD (2015). Report from the National Institute of Allergy and Infectious Diseases workshop on drug allergy. J Allergy Clin Immunol.

[32] Fontaine C, Mayorga C, Bousquet PJ, Arnoux B, Torres MJ, Blanca M (2007). Relevance of the determination of serum-specific IgE antibodies in the diagnosis of immediate beta-lactam allergy. Allergy.

[33] Blanca M, Mayorga C, Torres MJ, Reche M, Moya MC, Rodriguez JL (2001). Clinical evaluation of Pharmacia CAP System RAST FEIA amoxicilloyl and benzylpenicilloyl in patients with penicillin allergy. Allergy.

[34] Torres MJ, Romano A, Mayorga C, Moya MC, Guzman AE, Reche M (2001). Diagnostic evaluation of a large group of patients with immediate allergy to penicillins: the role of skin testing. Allergy.

[35] Sanz ML, Gamboa PM, Antepara I, Uasuf C, Vila L, Garcia-Aviles C (2002). Flow cytometric basophil activation test by detection of CD63 expression in patients with immediate-type reactions to betalactam antibiotics. Clin Exp Allergy.

[36] Sanz ML, Garcia BE, Prieto I, Tabar A, Oehling A (1996). Specific IgE determination in the diagnosis of beta-lactam allergy. J Investig Allergol Clin Immunol.

[37] Romano A, Mayorga C, Torres MJ, Artesani MC, Suau R, Sanchez F (2000). Immediate allergic reactions to cephalosporins: cross-reactivity and selective responses. J Allergy Clin Immunol.

[38] Antunez C, Blanca-Lopez N, Torres MJ, Mayorga C, Perez-Inestrosa E, Montanez MI (2006). Immediate allergic reactions to cephalosporins: evaluation of cross-reactivity with a panel of penicillins and cephalosporins. J Allergy Clin Immunol.

[39] Rouzaire P, Proton G, Bienvenu F, Guilloux L, Benoit Y, Piriou V (2012). IgE antibody detection in the diagnosis of hypersensitivity to neuromuscular blocking agents. Acta Anaesthesiol Scand.

[40] Laroche D, Chollet-Martin S, Leturgie P, Malzac L, Vergnaud MC, Neukirch C (2011). Evaluation of a new routine diagnostic test for immunoglobulin E sensitization to neuromuscular blocking agents. Anesthesiology.

[41] Ebo DG, Bridts CH, Hagendorens MM, Mertens CH, De Clerck LS, Stevens WJ (2006). Flow-assisted diagnostic management of anaphylaxis from rocuronium bromide. Allergy.

[42] Chung CH, Mirakhur B, Chan E, Le QT, Berlin J, Morse M (2008). Cetuximab-induced anaphylaxis and IgE specific for galactose-alpha-1,3-galactose. N Engl J Med.

[43] Mariotte D, Dupont B, Gervais R, Galais MP, Laroche D, Tranchant A (2011). Anti-cetuximab IgE ELISA for identification of patients at a high risk of cetuximab-induced anaphylaxis. MAbs.

[44] Garvey LH, Kroigaard M, Poulsen LK, Skov PS, Mosbech H, Venemalm L (2007). IgE-mediated allergy to chlorhexidine. J Allergy Clin Immunol.

[45] Macy E, Goldberg B, Poon KY (2010). Use of commercial anti-penicillin IgE fluorometric enzyme immunoassays to diagnose penicillin allergy. Ann Allergy Asthma Immunol.

[46] Johansson SG, Adedoyin J, van Hage M, Gronneberg R, Nopp A (2013). False-positive penicillin immunoassay: an unnoticed common problem. J Allergy Clin Immunol.

[47] Torres MJ, Padial A, Mayorga C, Fernandez T, Sanchez-Sabate E, Cornejo-Garcia JA (2004). The diagnostic interpretation of basophil activation test in immediate allergic reactions to betalactams. Clin Exp Allergy.

[48] Torres MJ, Ariza A, Mayorga C, Dona I, Blanca-Lopez N, Rondon C (2010). Clavulanic acid can be the component in amoxicillin-clavulanic acid responsible for immediate hypersensitivity reactions. J Allergy Clin Immunol.

[49] Sanchez-Morillas L, Perez-Ezquerra PR, Reano-Martos M, Laguna-Martinez JJ, Sanz ML, Martinez LM (2010). Selective allergic reactions to clavulanic acid: a report of 9 cases. J Allergy Clin Immunol.

[50] Longo N, Gamboa PM, Gastaminza G, Audicana MT, Antepara I, Jauregui I (2008). Diagnosis of clavulanic acid allergy using basophil activation and leukotriene release by basophils. J Investig Allergol Clin Immunol.

[51] Ebo DG, Leysen J, Mayorga C, Rozieres A, Knol EF, Terreehorst I (2011). The in vitro diagnosis of drug allergy: status and perspectives. Allergy.

[52] Uyttebroek AP, Sabato V, Leysen J, Bridts CH, De Clerck LS, Ebo DG (2014). Flowcytometric diagnosis of atracurium-induced anaphylaxis. Allergy.

[53] Gamboa PM, Sanz ML, Caballero MR, Antepara I, Urrutia I, Jauregui I (2003). Use of CD63 expression as a marker of in vitro basophil activation and leukotriene determination in metamizol allergic patients. Allergy.

[54] Gomez E, Blanca-Lopez N, Torres MJ, Requena G, Rondon C, Canto G (2009). Immunoglobulin E-mediated immediate allergic reactions to dipyrone: value of basophil activation test in the identification of patients. Clin Exp Allergy.

[55] Aranda A, Mayorga C, Ariza A, Dona I, Rosado A, Blanca-Lopez N (2011). In vitro evaluation of IgE-mediated hypersensitivity reactions to quinolones. Allergy.

[56] Ben Said B, Berard F, Bienvenu J, Nicolas JF, Rozieres A (2010). Usefulness of basophil activation tests for the diagnosis of IgE-mediated allergy to quinolones. Allergy.

[57] Rouzaire P, Nosbaum A, Denis L, Bienvenu F, Berard F, Cozon G (2012). Negativity of the basophil activation test in quinolone hypersensitivity: a breakthrough for provocation test decision-making. Int Arch Allergy Immunol.

[58] Kvedariene V, Kamey S, Ryckwaert Y, Rongier M, Bousquet J, Demoly P (2006). Diagnosis of neuromuscular blocking agent hypersensitivity reactions using cytofluorimetric analysis of basophils. Allergy.

[59] Fernandez TD, Torres MJ, Blanca-Lopez N, Rodriguez-Bada JL, Gomez E, Canto G (2009). Negativization rates of IgE radioimmunoassay and basophil activation test in immediate reactions to penicillins. Allergy.

[60] Sanz ML, Gamboa P, de Weck AL (2005). A new combined test with flowcytometric basophil activation and determination of sulfidoleukotrienes is useful for in vitro diagnosis of hypersensitivity to aspirin and other nonsteroidal anti-inflammatory drugs. Int Arch Allergy Immunol.

[61] Celik GE, Schroeder JT, Hamilton RG, Saini SS, Adkinson NF (2009). Effect of in vitro aspirin stimulation on basophils in patients with aspirin-exacerbated respiratory disease. Clin Exp Allergy.

[62] Bavbek S, Ikinciogullari A, Dursun AB, Guloglu D, Arikan M, Elhan AH (2009). Upregulation of CD63 or CD203c alone or in combination is not sensitive in the diagnosis of nonsteroidal anti-inflammatory drug intolerance. Int Arch Allergy Immunol.

[63] Ariza A, Fernandez TD, Dona I, Aranda A, Blanca-Lopez N, Melendez L (2014). Basophil activation after nonsteroidal anti-inflammatory drugs stimulation in patients with immediate hypersensitivity reactions to these drugs. Cytometry A.

[64] Ortega N, Dona I, Moreno E, Audicana MT, Barasona MJ, Berges-Gimeno MP (2014). Practical guidelines for diagnosing hypersensitivity reactions to nonsteroidal anti-inflammatory drugs. J Investig Allergol Clin Immunol.

[65] Mallal S, Phillips E, Carosi G, Molina JM, Workman C, Tomazic J (2008). HLA-B*5701 screening for hypersensitivity to abacavir. N Engl J Med.

[66] Phillips EJ, Chung WH, Mockenhaupt M, Roujeau JC, Mallal SA (2011). Drug hypersensitivity: pharmacogenetics and clinical syndromes. J Allergy Clin Immunol.

[67] Colombo S, Rauch A, Rotger M, Fellay J, Martinez R, Fux C (2008). The HCP5 single-nucleotide polymorphism: a simple screening tool for prediction of hypersensitivity reaction to abacavir. J Infect Dis.

[68] Chung WH, Hung SI, Hong HS, Hsih MS, Yang LC, Ho HC (2004). Medical genetics: a marker for Stevens–Johnson syndrome. Nature.

[69] Locharernkul C, Loplumlert J, Limotai C, Korkij W, Desudchit T, Tongkobpetch S (2008). Carbamazepine and phenytoin induced Stevens–Johnson syndrome is associated with HLA-B*1502 allele in Thai population. Epilepsia.

[70] Mehta TY, Prajapati LM, Mittal B, Joshi CG, Sheth JJ, Patel DB (2009). Association of HLA-B*1502 allele and carbamazepine-induced Stevens–Johnson syndrome among Indians. Indian J Dermatol Venereol Leprol.

[71] Tangamornsuksan W, Chaiyakunapruk N, Somkrua R, Lohitnavy M, Tassaneeyakul W (2013). Relationship between the HLA-B*1502 allele and carbamazepine-induced Stevens-Johnson syndrome and toxic epidermal necrolysis: a systematic review and meta-analysis. JAMA Dermatol.

[72] Pirmohamed M, Ostrov DA, Park BK (2015). New genetic findings lead the way to a better understanding of fundamental mechanisms of drug hypersensitivity. J Allergy Clin Immunol.

[73] Hung SI, Chung WH, Liou LB, Chu CC, Lin M, Huang HP (2005). HLA-B*5801 allele as a genetic marker for severe cutaneous adverse reactions caused by allopurinol. Proc Natl Acad Sci USA.

[74] Lonjou C, Borot N, Sekula P, Ledger N, Thomas L, Halevy S (2008). A European study of HLA-B in Stevens–Johnson syndrome and toxic epidermal necrolysis related to five high-risk drugs. Pharmacogenet Genomics.

[75] Lee MH, Stocker SL, Anderson J, Phillips EJ, Nolan D, Williams KM (2012). Initiating allopurinol therapy: do we need to know the patient’s human leucocyte antigen status?. Intern Med J.

[76] Mayorga C, Sanz ML, Gamboa P, Garcia-Aviles MC, Fernandez J, Torres MJ (2013). In vitro methods for diagnosing nonimmediate hypersensitivity reactions to drugs. J Investig Allergol Clin Immunol.

[77] Pichler WJ, Tilch J (2004). The lymphocyte transformation test in the diagnosis of drug hypersensitivity. Allergy.

[78] Zawodniak A, Lochmatter P, Yerly D, Kawabata T, Lerch M, Yawalkar N (2010). In vitro detection of cytotoxic T and NK cells in peripheral blood of patients with various drug-induced skin diseases. Allergy.

[79] Porebski G, Pecaric-Petkovic T, Groux-Keller M, Bosak M, Kawabata TT, Pichler WJ (2013). In vitro drug causality assessment in Stevens-Johnson syndrome—alternatives for lymphocyte transformation test. Clin Exp Allergy.

[80] Martin M, Wurpts G, Ott H, Baron JM, Erdmann S, Merk HF (2010). In vitro detection and characterization of drug hypersensitivity using flow cytometry. Allergy.

[81] Iammatteo M, Blumenthal KG, Saff R, Long AA, Banerji A (2014). Safety and outcomes of test doses for the evaluation of adverse drug reactions: a 5-year retrospective review. J Allergy Clin Immunol Pract.

